# The apolipoprotein E **ɛ**4 allele is no risk factor for prostate cancer in the Norwegian population

**DOI:** 10.1054/bjoc.2001.2098

**Published:** 2001-09-01

**Authors:** N Wessel, K Liestøl, J Mæhlen, S H Brorson

**Affiliations:** Department of Urology, Ullevål Hospital, Oslo, Norway; Department of Informatics, University of Oslo, Oslo, Norway; Department of Pathology, Ullevål Hospital, Oslo, Norway


*British Journal of Cancer* (2001) **85**(9), 1418
© 2001 Cancer Research Campaign
doi: 10.1054/ bjoc.2001.2098, available online at http://www.idealibrary.com on http://www.bjcancer.com

Letter to the Editor

### The apolipoprotein E ε4 allele is no risk factor for prostate cancer in the Norwegian population

**Sir,**

In 1998 a letter was published in the *British Journal of Cancer* (Lehrer et al, 1998) reporting increased frequency of the apolipoprotein E ε4 allele among patients with prostate cancer, and thereby suggests this allele as a risk factor for this type of cancer. Lehrer's study was based on examining 35 patients with prostate cancer. A study from Finland ($n$ = 130) (Niemi et al, 2000) did not show evidence that any ApoE-phenotype is associated with prostate cancer.

The study of Lehrer (1998) is one of many reports which claims association between ApoE and particular diseases. The ApoE ε4 allele is a strong risk factor for Alzheimer's disease (Strittmatter et al, 1993), and has been reported to be responsible for progression of atherosclerosis (Hixson et al, 1991), amyloidosis in patient with rheumatoid arthritis (Hasegawa et al, 1996), macro- and microangiopathy in diabetic patients (Ukkola et al, 1993) and decreased fertility (Gerdes et al, 1996). Finally, ApoE4 has been reported to have a protective effect against distal colon cancer (Kervinen et al., 1996).

It has been shown that the presence of ApoE has a protective effect on cells in culture due to an anti-oxidative effect, and that the ApoE2 has the largest protective effect, while ApoE4 is the least efficient phenotype for protecting cells from oxidative stress. Since the antioxidant vitamin E has been reported to have a protective effect against prostate cancer (Hartman et al, 1998), it seems reasonable to test the hypothesis that the ε4-allele is a risk factor for prostate cancer.

We have examined the hypothesis by comparing the distribution of apoE genotypes in 230 Norwegian patients (< 70 years) with prostate cancer to the distribution found in a recent study of 798 Norwegian blood donors (Kumar et al, submitted). ApoE-genotyping was carried out by polymerase chain reaction (Hixson and Vernier, 1990). The frequency of the ε4-allele in the patient population was 0.187, while the frequency of the ε4-allele in the control population was 0.198. This implies that there is no significant difference between the prostate cancer population and the normal population with respect to the frequency of the ε4-allele. The ε4/ε4-homozygosity appeared at a rate of 0.036 in the prostate cancer population, while a rate of 0.040 was observed for the normal Norwegian population (Kumar et al, submitted). This difference is also non-significant.

In conclusion, our data do not support the hypothesis that the ε4-allele is a risk factor for prostate cancer.

**N Wessel[1], K Liestøl[2], J Mæhlen[3] and SH Brorson[3]**

[1]Department of Urology, Ullevål Hospital, Oslo, Norway; [2]Department of Informatics, University of Oslo, Oslo, Norway; and [3]Department of Pathology, Ullevål Hospital, Oslo, Norway

### REFERENCES

Gerdes LU, Gerdes Chr, Hansen PS, Klausen Chr and Færgeman O (1996) Are men carrying the apolipoprotein E4 or E2 allele less fertile than e3e3 genotypes. *Hum Genet* **98**: 239–242

Hartman TJ, Albanes D, Pietinen P, Hartman AM, Rautalahti M, Tangrea JA and Taylor PR (1998) The association between baseline vitamin E, selenium, and prostate cancer in the alpha-tocopherol, beta-carotene cancer prevention study. *Cancer Epidemiol Biomarkers Prev* **7**: 335–340

Hasegawa H, Nishi S, Ito S, Saeki T, Kuroda T, Kimura H, Watababe T, Nakano M, Gejyo F and Arakawa M (1996) High prevalence of serum apolipoprotein E4 isoprotein in rheumatoid arthritis patients with amyloidosis. *Arthritis Rheum* **39**: 1728–1732

Hixson JE and PDAY Research group (1991) Apolipoprotein and progression of coronary artery disease evaluated by angiography and clinical events. *Circulation* **88**: 2763–2770

Hixson JE and Vernier DT (1990) Restriction isotyping of human apolipoprotein E by gene amplification and cleavage with Hhal. *J Lipid Res* **31**: 545–548

Kervinen K, Södervik H, Makela J, Lehtola J, Niemi M, Kairaluoma MI and Kesaniemi YA (1996) Is the Development of Adenoma and Carcinoma in Proximal Colon Related to Apolipoprotein E Phenotype? *Gastroenterology* **110**: 1785–1790

Kumar T, Liestøl K, Mæhlen J, Hiort A, Jettestuen E and Brorson SH. Allele frequencies of apolipoprotein E gene polymorphisms in the protein coding region and promotor region (-491 A/T) in a healthy Norwegian population. Human Biology. In press, 2001

Lehrer S (1998) Possible relationship of the apolipoprotein E (ApoE) e4 allele to prostate cancer. *Br J Cancer* **78**: 1398

Niemi M, Kervinen K, Kiviniemi H, Lukkarinen O, Kyllonen AP, Apaja-Sarkkinen M, Savolainen MJ, Kairaluoma Ml and Kesaniemi YA (2000) Apolipoprotein E phenotype, cholesterol and breast and prostate cancer. *J Epidemiol Community Health* **54**: 938–939

Strittmatter WJ, Saunders AM, Schmechel D, Pericak-Vance M, Enghild J, Salvesen GS and Roses AD (1993) Apolipoprotein E: high-avidity binding to beta-amyloid and increased frequency of type 4 allele in late-onset familial Alzheimer disease. *Proc Natl Acad Sci USA* **90**: 1977–1981

Ukkola O, Kervinen K, Salmela Pl, von Dickhoff K, Laakso M and Kesaniemi YA (1993) Apolipoprotein E phenotype is related to macro- and microangiopathy in patients with non-insulin-dependent diabetes mellitus. *Atherosclerosis* **101**: 9–15

